# Lexical Influences on Auditory Streaming

**DOI:** 10.1016/j.cub.2013.06.042

**Published:** 2013-08-19

**Authors:** Alexander J. Billig, Matthew H. Davis, John M. Deeks, Jolijn Monstrey, Robert P. Carlyon

**Affiliations:** 1 MRC Cognition and Brain Sciences Unit, 15 Chaucer Road, Cambridge, CB2 7EF, UK

## Abstract

Biologically salient sounds, including speech, are rarely heard in isolation. Our brains must therefore organize the input arising from multiple sources into separate “streams” and, in the case of speech, map the acoustic components of the target signal onto meaning. These auditory and linguistic processes have traditionally been considered to occur sequentially and are typically studied independently [1, 2]. However, evidence that streaming is modified or reset by attention [3], and that lexical knowledge can affect reports of speech sound identity [4, 5], suggests that higher-level factors may influence perceptual organization. In two experiments, listeners heard sequences of repeated words or acoustically matched nonwords. After several presentations, they reported that the initial /s/ sound in each syllable formed a separate stream; the percept then fluctuated between the streamed and fused states in a bistable manner. In addition to measuring these verbal transformations, we assessed streaming objectively by requiring listeners to detect occasional targets—syllables containing a gap after the initial /s/. Performance was better when streaming caused the syllables preceding the target to transform from words into nonwords, rather than from nonwords into words. Our results show that auditory stream formation is influenced not only by the acoustic properties of speech sounds, but also by higher-level processes involved in recognizing familiar words.

## Highlights

• Linguistic processing affects perceptual organization
• The acoustic elements of words fuse more readily than those of nonwords
• Bistable speech sounds share dynamics with other ambiguous perceptual objects

## Results

**Figure 1:**
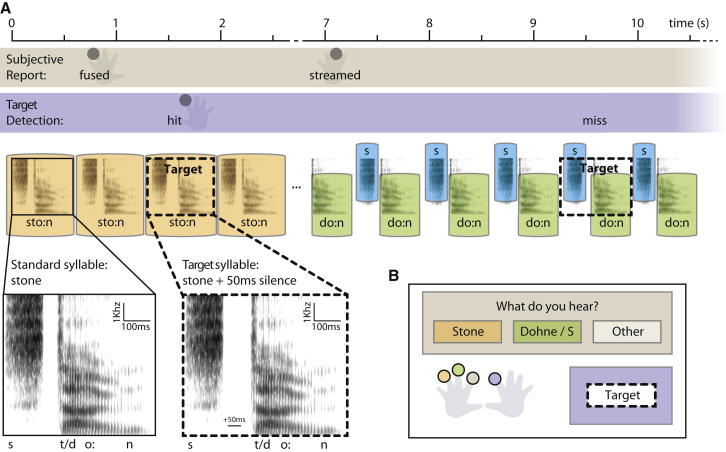
Experiment One Procedure (A) Schematic illustration of the time course of the start of one trial, in which the syllable is initially heard (and reported) as fused (“stone”) and then splits into two streams (“s” plus “dohne”). In this example, only the first of the two presented targets is detected. (B) Response interface in a trial where the syllable “stone” was presented. See also Figure S1.

On each trial in experiment one, we presented listeners with a single spoken syllable (either a real word, “stem” or “stone,” or a nonword, “stome” or “sten”) repeated every 672.5 ms for 150 s and asked them to report continuously what they heard (Figure 1). After a few seconds, the initial /s/ was reported to stream apart from the remainder of the sound and the percept then fluctuated in a bistable manner between the fused syllable and a two-stream percept of an isolated fricative /s/ plus the remainder of the syllable. This streaming, caused by differences in the spectral content of the two components of the syllable, transformed the words into nonwords (“dem” and “dohne”) and the nonwords into words (“dome” and “den”). (The lack of aspiration when a /t/ is preceded by /s/ leads to it being perceived as a /d/ when the /s/ is streamed apart [6]). The fluctuation between the two percepts is an example of a well-studied auditory illusion known as the verbal transformation effect (VTE) [5, 7–11].

**Figure 2:**
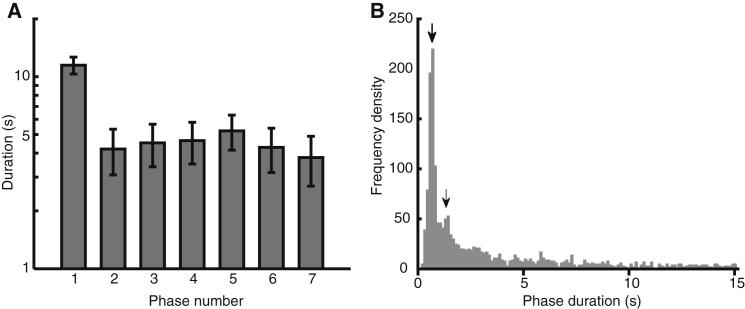
Experiment One Temporal Dynamics (A) Durations (on a log scale) of the first seven response phase durations. Data are drawn from the 12 participants who showed at least seven phases per trial. Error bars show the SEM, with across-participant differences removed (suitable for repeated-measures comparisons). (B) Distribution of response phase durations (excluding the first and last phases), based on data from all participants for all control trials (i.e., without targets) containing more than one phase. Arrows indicate peaks in the distribution at multiples of the stimulus onset asynchrony (672.5 ms).

Interestingly, the temporal dynamics of the observed switches in perceptual organization share several features with those previously observed not only for the buildup of streaming with pure tones, but also for certain bistable visual objects [12]: (1) the duration of the first (fused) percept was longer than that of subsequent percepts, all of which had about the same duration (Figure 2A), (2) the duration of each subsequent percept was independent of that of its predecessor (see the “Analyses” subsections in the Supplemental Experimental Procedures available online), and (3) the distribution of percept durations was negatively skewed (Figure 2B). Our results reinforce previous observations that bistability of speech and nonspeech sounds can arise from a common process, namely auditory streaming based on spectral differences [9], and provide further evidence for shared temporal dynamics across sound categories and sensory modalities [10, 12].

**Figure 3:**
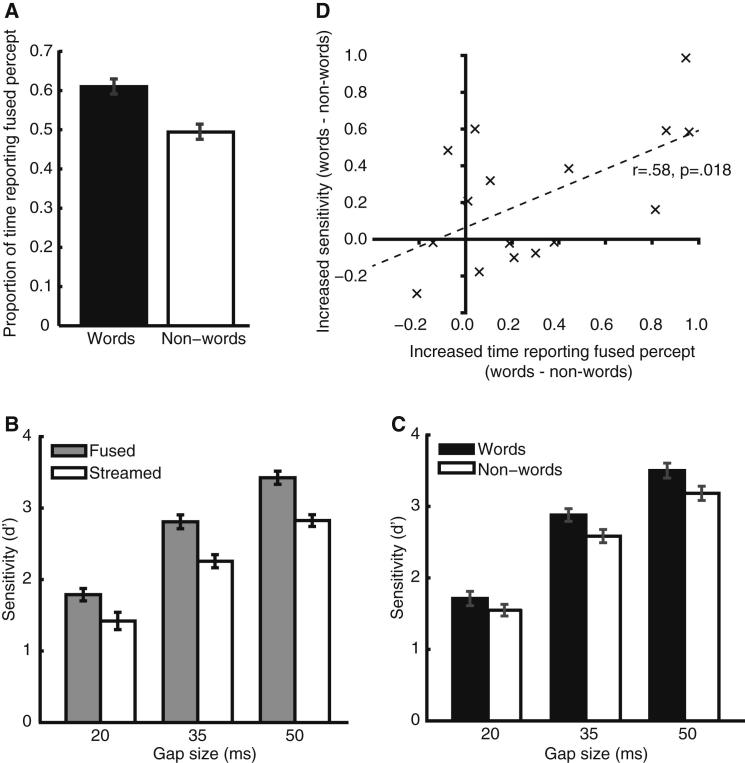
Experiment One Perceptual Report and Gap Detection Results Error bars show the SEM, with across-participant differences removed. (A) Proportion of time spent reporting the fused percept for stimuli where that form corresponded to a word or nonword. (B) Gap-detection sensitivity (*d*′) while reporting fused and streamed percepts. (C) Gap-detection sensitivity (*d*′) for syllables where the fused percept corresponded to a word or nonword, for each of the three gap sizes. (D) Between-participant correlation of the effects of lexicality on the time spent reporting the fused percept and on gap-detection sensitivity (averaged across gap sizes).

The amount of time that listeners spent reporting the fused percept was dependent on the linguistic content of the speech signal. Figure 3A shows that listeners reported the fused version of the syllable more frequently when it was a word than a nonword [repeated-measures ANOVA, F(1,15) = 9.31, p = 0.008; see the “Analyses” subsections in the Supplemental Experimental Procedures]. This is consistent with a previous observation that subjective reports of the VTE are altered by whether the repeated syllable forms a familiar word [5]. Here we can confirm that this apparent effect of lexical knowledge is not due to acoustic differences between words and nonwords: the stimuli were phonetically balanced (since the middle vowel and final consonant occurred equally often in words and nonwords) and acoustically well matched (Figure S1). This finding does not, however, show that lexical processing affects streaming per se; as with the subjective reports used in previous studies, it could simply be due to a postperceptual bias (whether conscious or not) to report hearing words over nonwords.

To rule out such a bias as an explanation of increased fused reports for words, we also required listeners to detect an additional silent gap inserted after the initial /s/ of a minority (6%) of syllables. The detection of this additional gap provides an objective and indirect measure of streaming, because listeners are poor at comparing the timing of sounds that have been segregated into different perceptual streams [13–15]. Figure 3B shows that gap detection was indeed better during phases where the listener reported the fused, compared to the streamed, percept [t(15) = 5.21, p < 0.001]. Crucially, performance was better when the fused syllable was a word than when it was a nonword, as predicted by an effect of word recognition on auditory streaming (repeated-measures ANOVA, F(1,15) = 8.87, p = 0.009; Figure 3C). Indeed, these two findings probably arose from the same processes: the effect of lexicality on reported streaming and on gap detection correlated significantly across listeners (r = 0.58, p = 0.018; Figure 3D).

**Figure 4:**
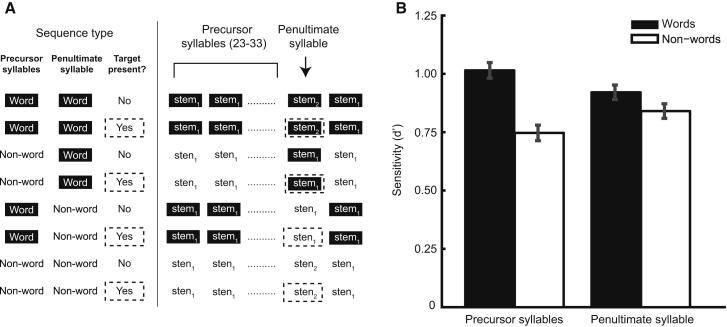
Experiment Two Design and Results (A) Eight example stimulus sequences, which factorially vary the lexical status of the precursor syllables and penultimate syllable, and the presence or absence of targets. The numerical subscripts indicate which of two different recordings of each syllable was presented, and targets are outlined with dotted lines. Note that an acoustic change always occurs at the penultimate syllable of a sequence, regardless of whether the lexical status is also different, or whether a target is present. (B) Gap-detection sensitivity (*d*′) for sequences in which the fused percept of the precursor syllables (left pair of bars) or penultimate syllable (right pair of bars) corresponded to a word or nonword. Error bars show the SEM, with across-participant differences removed. See also Figure S2.

However, an alternative explanation for the observed effect of lexicality on gap detection is that listeners have greater knowledge of the acoustic structure of words than nonwords from years of exposure to speech. These lexical templates might support the detection of acoustic irregularities (such as the additional gap in target syllables) without influencing streaming. Experiment two distinguished this account from the more theoretically important interpretation, in which linguistic knowledge affects perceptual organization. We presented a new group of listeners with 16–24 s sequences of the same syllables as in experiment one, recorded afresh by the same speaker. In half of these sequences, a gap was inserted between the /s/ of the penultimate syllable and its remainder to form a target. Importantly, we manipulated whether the penultimate syllable was a word or nonword independently of the other (“precursor”) syllables in each sequence (Figure 4A). Gap-detection performance was significantly better when the precursor syllables were words than when they were nonwords and was unaffected by whether the potential target was a word (repeated-measures ANOVA, main effect of precursor syllable lexicality [F(1,15) = 16.17, p = 0.001], no main effect of penultimate syllable lexicality [F(1,15) = 1.70, p = 0.212], no interaction [F(1,15) = 2.52, p = 0.133]; Figure 4B). This demonstrates that the lexical status of the sequence of precursor syllables affects the buildup of streaming and is inconsistent with the effects reported here being due to listeners using a lexical template to detect the target. As a further control, we presented listeners with isolated versions of each syllable in a two-alternative forced-choice adaptive task and found no effect of lexicality on gap detection [t(15) = 0.28, p = 0.780 for experiment one tokens; t(15) = 1.56, p = 0.139 for experiment two tokens; see the Supplemental Experimental Procedures].

## Discussion

The neural and cognitive basis of auditory streaming remains an issue of intense debate. Correlates of the streaming of pure tones have been observed in the intraparietal sulcus [16], auditory cortex [17, 18], and even in the brainstem [19]. In these latter two cases, it is possible to account for the dependence of streaming on the physical aspects of the stimulus in terms of the passive response properties of individual neurons. The present study investigated the extent to which these “primitive” streaming mechanisms are modified by higher-level processes involved in the perception of speech.

It is well known that training and experience can affect the reports that listeners make when presented with a speech sound. There are several ways in which this can occur, not all of which demonstrate an effect on primitive streaming processes. For example, in our experiment one, and in previous investigations of the VTE using subjective measures [5, 8], listeners had a greater tendency to report fused percepts that corresponded to words than to nonwords. This could be due to a response bias. A subtler distinction arises when knowledge helps the listener to interpret ambiguous sensory information. For example, degraded speech sounds are heard as more speech-like when the listener is told in advance what the message is [20] or, in some cases, even that it is speech [21]. For some stimuli, this disambiguation may affect the perceived fusion of the stimulus [21]; for others, however, the primary effect may not be on fusion but on the intelligibility of a degraded utterance [20]. It may be viewed as a case of what Bregman [1] refers to as “schema-based” processing. This can be distinguished from primitive stream segregation in at least two ways. First, familiarity with a target stimulus within a mixture does not help identification of the background [22]. Second, it may give the listener access to more than one perceptual organization. An example comes from one type of degraded speech, known as “sine-wave speech”: telling listeners that the stimulus is speech causes them to report hearing the formant-tracking sine waves as fused (giving rise to a phonetic percept), but they can still identify individual components within the mixture [23]. There is no evidence that knowledge that the stimulus is speech aids segregation from a competing source.

We argue that the strength of evidence that a particular manipulation affects primitive streaming depends on at least two aspects of the measurements obtained. First, objective tasks should be preferred over subjective reports, not only because the latter may reflect response biases, but also because they may arise from a voluntary parsing of a sensory representation that occurs at the output of primitive streaming processes. Second, as several authors have pointed out [3, 4, 24], it is preferable to assess perception using a stimulus that is different from that on which the knowledge, training, or—as in our case—lexical manipulation is applied. Both of our experiments meet the first criterion, and experiment two—in which we show that lexicality of the precursors affects detection of gaps in the targets (irrespective of whether the targets are words or nonwords)—meets the second one.

The verbal transformations that we have studied here provide a striking example of how the brain evaluates and switches between alternative interpretations of ambiguous sensory input. Although these transformations are induced by the spectral characteristics of natural speech, we demonstrate that the primitive mechanisms involved in forming these percepts are influenced by word knowledge. Our results may inform future studies of the neural basis of these top-down effects [10, 25], which naturally suggest accounts in which high-level knowledge serves to predict acoustic input [20, 26]. Whereas models of auditory streaming and of spoken word recognition are usually developed in isolation, our findings provide an empirical foundation for more comprehensive accounts that incorporate reciprocal interactions between these two important aspects of speech perception.

## Experimental Procedures

Please refer to the Supplemental Experimental Procedures for additional detail. All experimental procedures were approved by the Cambridge Psychology Research Ethics Committee.

### Experiment One

#### Participants

We tested 17 native English speakers with normal hearing and discarded the data of one participant who could not perform the gap detection task reliably.

#### Stimuli

The syllables “stem,” “sten,” “stone,” and “stome” were recorded by a speaker of southern British English. The initial /s/ was excised from each syllable, and the /s/ from “stone” was cross-spliced with the remainder (“dem,” “den,” “dome,” and “dohne”) of each syllable. Prior to this cross-splicing, the duration of the /s/ and of each “remainder” was reduced, such that the reconstructed syllables could be presented at a rate sufficiently fast to support streaming [27]. The intensities of the shortened /s/ and of the shortened remainders were scaled to the same root-mean-square value, and the components then recombined. For the “standard” stimuli, the silent gap between the initial /s/ and the rest of the syllable was 50 ms, comparable to the duration of the silent closure in the original recording. For the target sounds, an additional silent gap of 20, 35, or 50 ms was inserted. Waveforms for the standard stimuli in both experiments are shown in Figure S2 (stimuli and example sequences can be downloaded as .wav files at http://www.mrc-cbu.cam.ac.uk/people/alex-billig/lexical-streaming/). All stimuli were presented diotically over Sennheiser HD650 headphones at a level of 64 dB second pressure level to listeners seated in a double-walled sound-insulating room.

#### Task

The session started with a series of practice trials to introduce the VTE and the report and detection tasks. Each trial in the main experiment consisted of 223 repetitions of one of the syllables “stem,” “sten,” “stome,” or “stone,” with a stimulus onset asynchrony (SOA; time between onsets of successive /s/ sounds) of 672.5 ms. These were presented in blocks of four sequences, one for each syllable. The first block consisted of control trials, which contained no target syllables. Participants were required to indicate the percept being heard, using one of three keys on a computer keyboard (linked to a display of current response options, e.g., “Stem,” “Dem / S,” or “Other”). They were also told to adjust their response as required throughout the sequence.

After the control trials, further blocks of four sequences followed with targets having 50 ms, then 35 ms, then 20 ms additional gaps. These three blocks were then repeated, giving a total of two experimental trials for each combination of token and gap size. A random number of between 11 and 17 standard stimuli were presented between successive targets, and no targets were presented after the 217^th^ syllable in each sequence. These parameters led to approximately 6% targets in each of the experimental trials. In addition to reporting their percept throughout the experimental trials, participants indicated their detection of any targets with another keystroke.

#### Analyses

Target responses were scored as hits if they occurred within 1.435 s of the start of a target and as misses otherwise. The data for each syllable and gap size were then converted to sensitivity (*d*′) scores and analyzed using repeated-measures ANOVAs.

### Experiment Two

#### Participants

We tested 16 native English speakers with normal hearing, none of whom had taken part in experiment one.

#### Stimuli

Two new recordings of each syllable from experiment one were made by the same speaker such that syllables were sufficiently short to be presented with a SOA of 672.5 ms at their natural durations The initial /s/ was excised from each syllable, and the /s/ from one of the “stem” tokens was cross-spliced with the remainder of each syllable. Intensity normalization was applied to the set of cross-spliced syllables. Targets were created by insertion of an additional 40 ms of silence between the /s/ and the remainder of the syllable.

#### Task

Participants were introduced to the VTE and the concept of auditory streaming, and practiced the two components of the task (described below). Each trial in the main experiment consisted of 25, 27, 29, 30, 31, 33, or 35 syllables presented with an SOA of 672.5 ms. The penultimate syllable in each sequence was always a different token from the other syllables in the sequence, which were all identical. For each of the eight precursor tokens (two different recordings of the four syllables “stem,” “sten,” “stome,” and “stone”), half of the sequences used the other token of the same syllable in the penultimate position. The penultimate syllable in the other half of the sequences used the token with the same first vowel sound but a different final consonant (e.g., “stome” for “stone”; see Figure 4A). Thus, the penultimate syllable always differed acoustically from the precursor syllables. In each of these 16 conditions, half of the sequences had a target in the penultimate position. Each participant heard 224 sequences (one for each combination of sequence length, precursor token, penultimate token, and target presence) presented in eight blocks of 28 sequences.

Participants were required to indicate the percept being heard using one of three keys on a computer keyboard (linked to a display of response options, e.g., “One stream,” “Two streams,” or “Other”). They were also told to adjust their response as required throughout the sequence. After the sequence ended, participants were asked, “Was the gap after the penultimate ‘s’ longer?” and selected one of four responses labeled “Definitely,” “Probably,” “Probably Not,” and “Definitely Not.”

#### Analyses

“Definitely” or “Probably” responses were scored as hits if they occurred at the end of a sequence in which a target was present in the penultimate position, and as false alarms otherwise. The data for each combination of precursor and penultimate token were converted to sensitivity (*d*′) scores and analyzed using repeated-measures ANOVAs.

## Supplemental Information

Document S1. Supplemental Experimental Procedures and Figures S1 and S2
