# Supplementary material for: Lexical Influences on Auditory Streaming
Source: Curr Biol. 2013 Aug 19;23(16):1585–9. doi: 10.1016/j.cub.2013.06.042 (PMC3748342; doi:10.1016/j.cub.2013.06.042)
Supplement: Document S1. Supplemental Experimental Procedures and Figures S1 and S2 [file mmc1.pdf]

Current Biology, Volume *23*

Supplemental Information

Lexical Influences on Auditory Streaming

Alexander J. Billig, Matthew H. Davis, John M. Deeks, Jolijn Monstrey, and Robert P. Carlyon

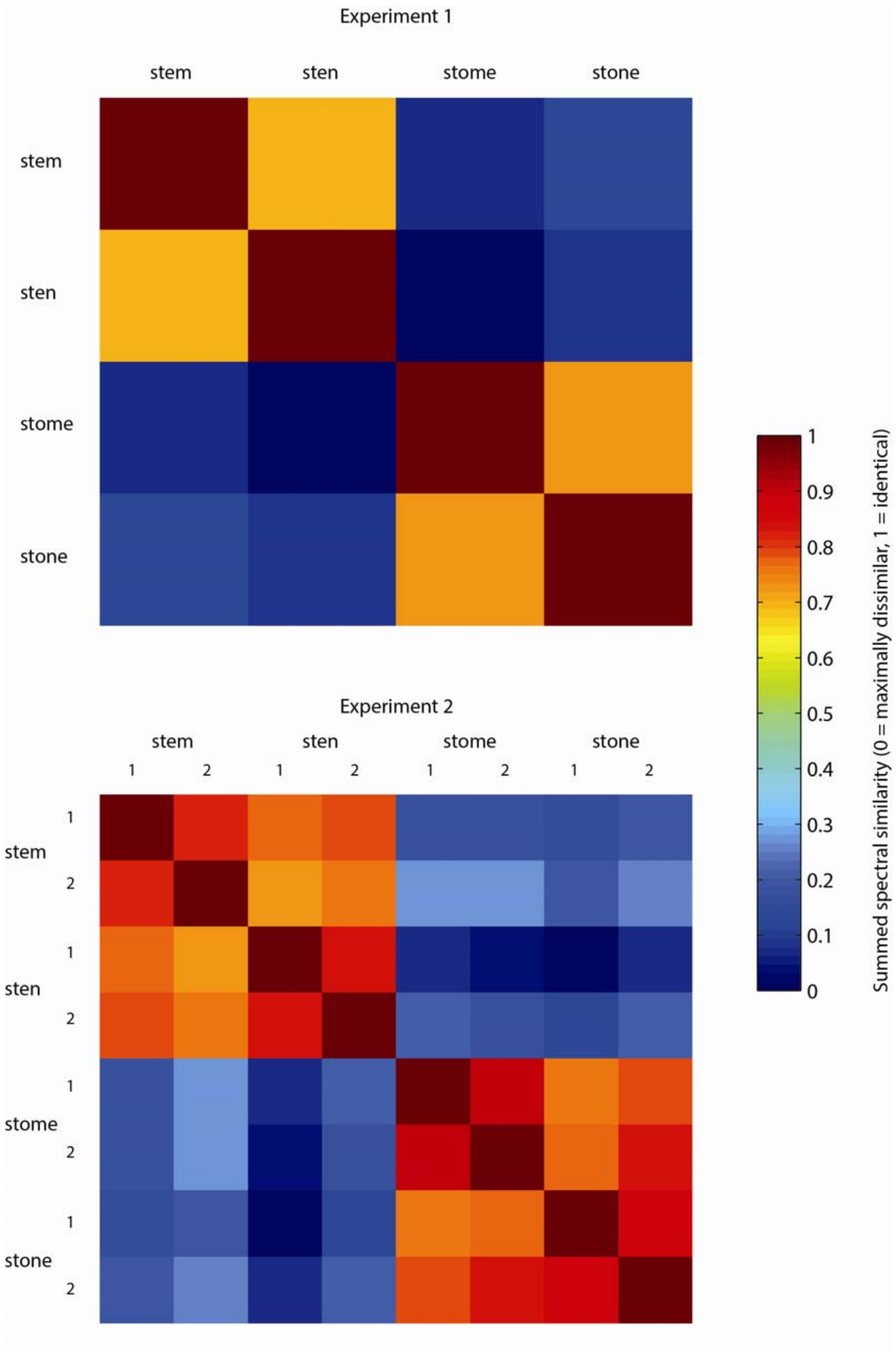

Experiment 1
stem
sten
stome
stone
Experiment 2
1
2
Summed spectral similarity (0 = maximally dissimilar, 1 = identical)
0
0.1
0.2
0.3
0.4
0.5
0.6
0.7
0.8
0.9
1

Figure S1. Related to Figure 1. Matrices illustrating the spectro-temporal similarity between stimuli, for Experiment One (top) and Experiment Two (bottom). For each token, a Gammatone-based Fourier transform was computed, approximating the frequency analysis performed by the ear. A spectral similarity matrix was then generated for each pair of tokens in an experiment by comparing the transform output (on a log scale) across all time slices. Next, the maximum-similarity path through this matrix was found using dynamic time warping. Summed similarity values along this path were computed and rescaled (1 – similarity / maximum similarity) such that two identical sound files were assigned a score of 1 and the two most dissimilar sound files given a score of 0. Note that greatest similarity (toward the red end of the spectrum) is seen for pairs of syllables that have the same vowels, rather than for syllables that have the same lexical status (words – “stem”/“stone”, and non-words – “sten”/“stome”). The transform and dynamic time warping were performed based on code by Ellis, available at http://www.ee.columbia.edu/~dpwe/resources/matlab/gammatonegram/ and http://labrosa.ee.columbia.edu/matlab/dtw.

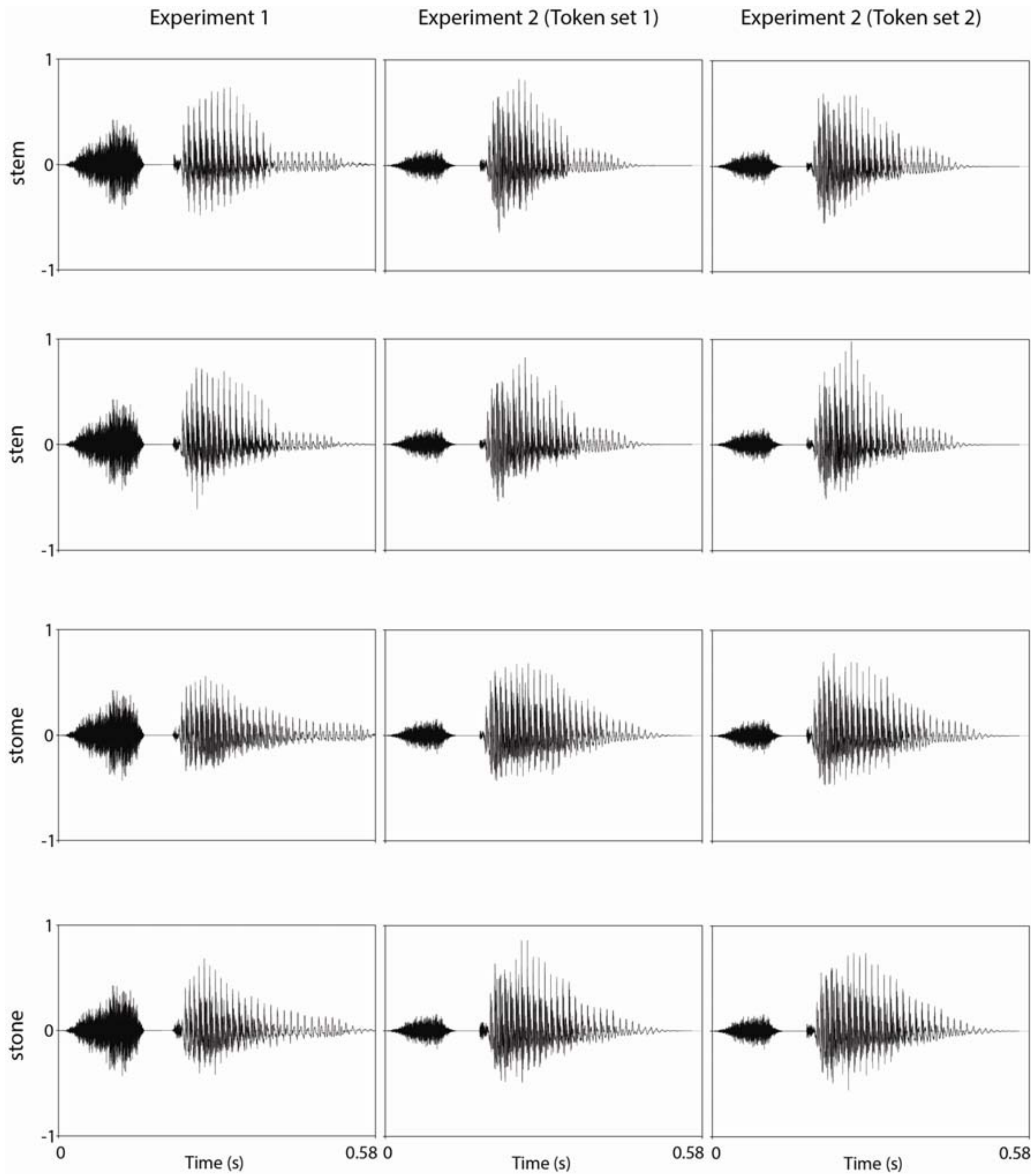

Figure S2. Related to Figure 4. Waveforms for the standard stimuli in Experiment One (first column) and Experiment Two (second and third columns). The variation across experiments in the intensity of the /s/ segments relative to that of the syllable remainders results from differences in the normalization processes (see Supplemental Experimental Procedures), but all tokens retained a natural speech quality.

Supplemental Experimental Procedures

Experiment One

Participants

We tested 17 native English speakers and discarded the data of one participant who could not perform the gap detection task reliably (*d'* for the easiest 50-ms gap targets was 0.8, compared to mean of 3.4 for other listeners). The average pure-tone threshold across 0.5, 1, 2, 4 kHz was < 10 dB HL for each of the final 16 participants. All experimental procedures were approved by the Cambridge Psychology Research Ethics Committee.

Stimuli

The syllables “stem”, “sten”, “stone”, and “stome” were recorded by a speaker of southern British English (author MHD) in a double-walled sound-insulated room using a Marantz PMD670 portable solid state recorder (44.1 kHz sampling rate, 16 bit resolution) and AKG C1000S microphone. Subsequent analysis and processing was performed in Adobe Audition 3.0. The initial /s/ was excised from each syllable, and the /s/ from “stone” was cross-spliced with the remainder (“dem”, “den”, “dome” and “dohne”) of each syllable. Prior to this cross-splicing, the duration of the /s/ and of each “remainder” was reduced by removing non-adjacent cycles at their zero-crossings, such that the reconstructed syllables could be presented at a rate sufficiently fast to support streaming [1]. It reduced the duration of the /s/ from 216 to 158 ms, and ensured that each of the syllable remainders had the same duration of 375 ms (a reduction in duration of 137-192 ms). The intensities of the shortened /s/ and of the shortened remainders were scaled to the same root mean-square (RMS) value, and the components then recombined. For the “standard” stimuli the silent gap between the initial /s/ and the rest of the syllable was 50 ms, comparable to the duration of the silent closure in the original recording. For the target sounds an additional silent gap of 20, 35, or 50

ms was inserted. Waveforms for the standard stimuli are shown in Fig. S2 (stimuli and example sequences can be downloaded as .wav files at http://www.mrc-cbu.cam.ac.uk/people/alex-billig/lexical-streaming/). Fig. S1 shows the results of a spectro-temporal analysis demonstrating that the acoustic similarity between the syllables was primarily driven by vowel identity and not by lexical status. The fundamental frequency of the voiced portions of the syllables was, on average, 88 Hz and did not differ significantly between words and non-words. Practice trials (see below) used the syllable “stope” spoken by another speaker of Southern British English (author AJB) and processed in the same way. All stimuli were presented diotically over Sennheiser HD650 headphones at a level of 64 dB SPL, to listeners seated in a double-walled sound-insulating room.

Task

The session started with a series of practice trials to introduce the VTE and the report and detection tasks. Participants first heard a one-minute repeating sequence of the syllable “stope” during which they were familiarized with the phenomenon of the VTE and hence that the percept might occasionally change during the presentation. They then heard another one-minute sequence during which they pressed one of three keys on a computer keyboard (linked to a visual display of current response options, e.g. “Stem”, “Dem / S”, or “Other”) to indicate which percept they were hearing. They were also told to adjust their response as required throughout the sequence. The fused stimulus was highlighted by default at the beginning of the sequence, in both the practice and the experimental trials. Next, participants were alerted to the possible presence of targets and performed a further one-minute practice, during which they were required to detect occasional targets with an additional 50-ms gap. Finally, they heard a 2.5-minute sequence during which they performed both tasks concurrently.

The experiment proper began with a block of four control trials (one for each of “stem”, “sten”, “stome” and “stone”) without targets; participants were only required to indicate the

percept being heard. Each sequence in the main experiment consisted of 223 syllables with a stimulus onset asynchrony ("SOA"; time between onsets of successive /s/ sounds) of 672.5 ms. The order of syllables used within a block was counterbalanced across participants with a Latin square design. This order remained constant for each participant throughout the experiment, being used in each subsequent block of four sequences. After the control trials, further blocks of four sequences followed with targets having 50-ms, then 35-ms, then 20-ms additional gaps. These three blocks were then repeated, giving a total of two experimental trials for each combination of token and gap size. A random number of between 11 and 17 standard stimuli were presented between successive targets, and no targets were presented after the 217th syllable in each sequence. These parameters led to approximately 6% targets in each of the experimental trials. Participants were encouraged to take breaks between blocks. The entire procedure (including training, breaks and audiometric testing) lasted approximately three hours.

Analyses

Target responses were scored as hits if they occurred within 1.435 s of the start of a target and as misses otherwise. The 1.435-s cutoff was chosen as the latency within which 95% of all responses were made to the easiest (50-ms) targets, pooled across all syllables and participants. The data for each syllable and gap size were then converted to sensitivity (*d'*) and criterion scores and analyzed using repeated measures ANOVAs. Criterion values did not differ across conditions and are not reported. The proportion of time spent in the fused percept was *z*-transformed for each syllable before being analyzed using a repeated measures ANOVA.

"Other" percepts accounted for 9.2% of total trial time across participants and were significantly more frequent when the fused syllable was a non-word than when it was a word ($t(15)=2.39$, $p=.030$). The analysis of gap detection performance by lexicality was based on

all syllables in all trials, but the same pattern of results holds when excluding syllables for which “other” percepts were reported.

To assess the independence of the durations of successive percepts (“phases”), we performed the same analysis as in [2], using the four “control” trials with no targets, and with the first and last phases of each trial excluded. For the 15 participants with a sufficiently high number of phases per trial, we calculated the correlation between the log-transformed duration of each phase and that of its predecessor. This correlation did not differ significantly from zero (Wilcoxon signed-rank test, p=.302). The distribution of phase durations (histogram in Fig. 2b) resembles the log-normal distribution observed previously for tones and visual objects [2]. It deviates slightly from that distribution for a trivial reason: because listeners assessed their percept based on whether or not each syllable was streamed, there are peaks in the distribution at integer multiples of the SOA (672.5 ms; arrows in Fig. 2b).

Another explanation for better target detection during sequences of words than of non-words is that lexicality only affects participants’ streaming *reports* (rather than *perception*), but that the percept-reporting process interferes with gap detection. This hypothesis would predict that the “undisturbed period” preceding each target (i.e. the elapsed time between that target and the previous percept report, or – if no percept reports have yet occurred in the trial – between that target and the start of the trial) should be greater (a) when the syllables heard during that period were words than when they were non-words, and (b) when the target was detected than when it was missed. This was not the case (lexicality: $t(15)=1.11$, p=.285; hit/miss: $t(15)=1.87$, p=.081 in opposite direction from that predicted).

Experiment Two

Participants

We tested 16 native English speakers, none of whom had taken part in Experiment One. The average pure-tone threshold across 0.5, 1, 2, 4 kHz was < 15 dB HL for each of the participants. All experimental procedures were approved by the Cambridge Psychology Research Ethics Committee.

Stimuli

Two new recordings of each syllable from Experiment One were made by the same speaker using a metronome during recording to ensure that syllables were sufficiently short to be presented with a SOA of 672.5 ms at their natural durations. The initial /s/ was excised from each syllable, and the /s/ from one of the “stem” tokens was cross-spliced with the remainder of each syllable. Intensity normalization was applied to the set of cross-spliced syllables. Targets were created by inserting an additional 40 ms of silence between the /s/ (with a duration of 172 ms including natural silence) and the remainder of the syllable (with a duration of 389 ms). Waveforms for the standard stimuli are shown in Fig. S2 (stimuli and example sequences can be downloaded as .wav files at http://www.mrc-cbu.cam.ac.uk/people/alex-billig/lexical-streaming/). Fig. S1 shows the results of a spectro-temporal analysis demonstrating that the acoustic similarity between the syllables was primarily driven by vowel identity and not by lexical status. The fundamental frequency of the voiced portions of the syllables was, on average, 89 Hz and did not differ significantly between words and non-words. A recording of the syllable “sti” was made by the same speaker, processed in the same way, and used in the pre-experiment familiarization (see below).

Task

Participants first heard a one-minute repeating sequence of the syllable “sti” during which they were introduced to the phenomenon of the VTE, as in Experiment One. The concept of auditory streaming was also outlined, and it was verified that participants could experience both a one-stream percept (a single sequence of repeated “sti” sounds) and a two-stream percept (two simultaneous sequences of repeated sounds – “s” / “di” – with one sequence possibly sounding more “in the foreground” than the other). They then completed practice trials with the syllable “stope” for the two components of the task (described below; practice was for each component separately, then combined). Each sequence in the main experiment consisted of either 25, 27, 29, 30, 31, 33 or 35 syllables, presented with an SOA of 672.5 ms. The penultimate syllable in each sequence was always a different token from the other syllables in the sequence, which were all identical. For each of the eight precursor tokens (two different recordings of the four syllables “stem”, “sten”, “stome”, “stone”), half of the sequences used the other token of the same syllable in the penultimate position. The penultimate syllable in the other half of the sequences used the token with the same first vowel sound, but a different final consonant (e.g. “stome” for “stone”; see Fig. 4a). Thus, the penultimate syllable always differed acoustically from the precursor syllables. In each of these 16 conditions, half of the sequences had a target in the penultimate position. Each participant heard 224 sequences, one for each combination of sequence length (7 conditions), precursor token (8 conditions), penultimate token (2 conditions) and target presence (2 conditions). These were presented in eight blocks of 28 sequences. The first 14 sequences in a block used the same precursor as each other; the precursors in the remaining sequences used the other token of the same syllable. Subject to this constraint, sequences in a block were picked at random and the assignment of precursor syllables to blocks was counterbalanced across participants with a Latin Square design.

At the beginning of each sequence, the question “What do you hear?” was displayed at the top of the screen with three percept response keys. These were labelled as “One stream”, “Two streams”, and “Other” and remained so through the experiment. This differed from Experiment One (where listeners reported the item they were hearing, e.g. “stem” versus “dem / s”) as the presented syllable changed (e.g. from “stem” to “sten”) at the penultimate position on 50% of trials. None of these responses was initially highlighted and participants were instructed to indicate what they heard as soon as possible, and every time their percept changed. If no percept response was made during the entire sequence, a message reminded participants of the task requirements, and the sequence was discarded (and re-presented later in the experiment).

After the sequence ended, participants were asked: “Was the gap after the penultimate ‘s’ longer?”, and given unlimited time to select one of four responses labelled: “Definitely”, “Probably”, “Probably Not”, and “Definitely Not”. Participants were reminded to base their judgment solely on the size of the gap between the /s/ and the remainder of each syllable, and not on other acoustic differences. They were also told to give the two tasks (percept report and gap detection) equal priority. As soon as a response had been made about the presence of a gap in the penultimate syllable, the next sequence in a block began. Participants were encouraged to take breaks between blocks.

During the same session, participants’ sensitivity to gaps in isolated tokens was tested with a two-alternative forced-choice task. Trials consisted of pairs of stimuli, presented in intervals separated by 500 ms of silence. One interval (selected at random for each trial) contained a syllable with additional silence inserted between the /s/ and the remainder (similar to targets in Experiments One and Two), while the other interval contained the same syllable without additional silence. After each pair of stimuli, participants indicated which interval they believed contained the syllable with the additional gap, the size of which varied

adaptively from trial to trial. The next pair of stimuli were presented 200 ms after a response. Participants completed two adaptive staircases for each of the eight tokens from Experiment Two (at the start of the testing session), and for each of the four tokens from Experiment One (at the end of the testing session), with the order of tokens counterbalanced using a Latin Square design. The size of the additional gap was 100 ms at the beginning of each staircase; it decreased following two consecutive correct responses and increased following each incorrect response (tracking the 70.7% correct point on the psychometric function). The gap size was scaled in the appropriate direction by a factor of 1.4 until two reversals had occurred, and by a factor of 1.1 for the following six reversals, after which the staircase ended. Before testing, each participant completed a practice staircase using the syllable “stope” from Experiment Two. The entire procedure (main experiment, adaptive staircases, training, breaks and audiometric testing) lasted approximately three hours.

Analyses

“Definitely” or “Probably” responses were scored as hits if they occurred at the end of a sequence in which a target was present in the penultimate position, and as false alarms otherwise. The data for each combination of precursor and penultimate token were converted to sensitivity (*d’*) and criterion scores and analyzed using repeated measures ANOVAs. As the sensitivity and criterion scores were not normally distributed, additional non-parametric signed ranks tests were carried out; these showed the same pattern of results. The proportion of penultimate syllables reported as one stream was *z*-transformed for each token before being analyzed using a repeated measures ANOVA.

Across participants, “other” percepts were reported for 2.2% of penultimate syllables. In 0.3% of trials, no percept had been reported by the penultimate syllable. Neither of these proportions differed significantly between sequences with word versus non-word precursor syllables (“other” percepts: $t(15)=0.32$, $p=.751$; no percept: $t(15)=0.29$, $p=.774$). The analysis

of gap detection performance by lexicality of precursor/penultimate syllables was based on all trials, but the same pattern of results holds when excluding trials in which no percept or an "other" percept was reported for the penultimate syllable.

The "undisturbed" period preceding targets (see Supplemental Experimental Procedures – Experiment One – Analyses for details) was no greater for word than for non-word precursor syllables ($t(15)=1.39$, $p=.185$), and no greater for hits than for misses ($t(15)=0.26$, $p=.799$).

It is a priori possible that, when there is a large acoustic difference between the precursors and the penultimate syllable, streaming may be reset, causing the penultimate syllable to be heard as fused and leading to better gap detection performance. If this were the case, performance would be expected to be better when the lexicality of the precursors and penultimate syllable differed, compared to when it matches. Although this could not alone explain the observed main effect of precursor lexicality on performance, we report d' scores for each combination of precursor and penultimate syllable lexicality for completeness: WW=1.15, WN=0.88, NN=0.80, NW=0.70 (first letter indicates precursor lexicality, second letter indicates penultimate syllable lexicality, W=Word, N=Non-word). Note in particular that performance is numerically worse ($t(15)=1.59$, $p=.133$) when the lexicality changes for the penultimate syllable than when it does not, which is inconsistent with the hypothesis.

For the two-alternative forced-choice task, the gap sizes at each of the last six reversal points were averaged for each staircase. These thresholds were collapsed across tokens of the same lexicality, and analyzed separately for each experiment using paired *t*-tests.

Supplemental References

1. Anstis, S., and Saida, S. (1985). Adaptation to auditory streaming of frequency modulated tones. J. Exp. Psychol. Hum. Percept. Perform. *11*, 257-271.
2. Pressnitzer, D., and Hupé, J.-M. (2006). Temporal dynamics of auditory and visual bistability reveal common principles of perceptual organization. Curr. Biol. *16*, 1351-1357.
